# High-Quality Draft Genome Sequence of Sphaerisporangium cinnabarinum ATCC 31213

**DOI:** 10.1128/genomeA.00456-18

**Published:** 2018-05-24

**Authors:** Line Friis Bakmann Christensen, Daniel Otzen, Morten Simonsen Dueholm

**Affiliations:** a Department of Molecular Biology, Interdisciplinary Nanoscience Center (iNANO), Aarhus University, Aarhus, Denmark; b Department of Chemistry and Bioscience, Center for Microbial Communities, Aalborg University, Aalborg, Denmark

## Abstract

A high-quality draft genome sequence of Sphaerisporangium cinnabarinum ATCC 31213 is presented here. This bacterium produces several important bioactive compounds and may also produce functional amyloids. This is the first sequenced genome from the genus Sphaerisporangium, and it will be essential in determining the nature of the potential amyloid protein.

## GENOME ANNOUNCEMENT

Members of the genus Sphaerisporangium are aerobic Gram-positive bacteria that form mycelia and spherical sporangia (1). They are primarily found in soils (2, 3), often in association with the rhizosphere (4–7). Many members of the genus produce bioactive secondary metabolites (8–12) and can also be used for the biotransformation of antimicrobials (13).

Sphaerisporangium cinnabarinum (previously referred to as Streptosporangium cinnabarinum) strain ATCC 31213 was originally isolated in 1977 from soil in the Philippines and was shown to produce two different antibiotics effective against strains of Staphylococcus aureus, Mycobacterium smegmatis, and Bacillus subtilis (14). Another antibiotic produced by S. cinnabarinum, the secondary metabolite GE82832, specifically inhibits translation in bacteria (10–12). S. cinnabarinum also produces 1-hydroxy-4-methoxy-2-naphthoic acid, which is an effective herbicide against the freshwater plant Lemna minor (15). Recently, S. cinnabarinum was proposed to form functional amyloids, as the mycelia showed strong binding to conformation-specific antibodies that recognize a generic amyloid fibril epitope (16, 17). Further studies of S. cinnabarinum, including identification of the proposed amyloid protein and identification of pathways for antibiotic synthesis, would benefit from this genome announcement.

Genomic DNA was isolated using a FastDNA spin kit (MP Biomedicals). A paired-end library was prepared with the NEBNext Ultra II DNA library prep kit for Illumina (New England BioLabs). All procedures were carried out as recommended by the manufacturer. Sequencing of the libraries was performed using a MiSeq sequencer (Illumina, Germany). The paired-end reads were trimmed for adapters and quality using the built-in tool of CLC Genomics Workbench version 9.5.5 (Qiagen, USA). The genome was *de novo* assembled from the paired-end data in CLC Genomics Workbench using the default settings. The average coverage of the assembly was 60×. Annotation was done using the NCBI Prokaryotic Genome Automatic Annotation Pipeline (PGAAP) (18).

The draft genome sequence of S. cinnabarinum strain ATCC 31213 is composed of 134 contigs and has a predicted size of ∼4.39 Mbp. The overall G+C content is 74.7%. Annotation by the NCBI PGAAP identified 3,988 coding sequences (CDSs), as well as 3 rRNA (5S, 16S, or 23S) and 51 tRNA genes.

### Accession number(s).

This whole-genome shotgun project has been deposited at DDBJ/EMBL/GenBank under the accession no. QAPD00000000. The version described in this paper is the first version, QAPD01000000.
